# A bihemispheric autonomic model for traumatic stress effects on health and behavior

**DOI:** 10.3389/fpsyg.2014.00843

**Published:** 2014-08-01

**Authors:** Sung W. Lee, Lee Gerdes, Catherine L. Tegeler, Hossam A. Shaltout, Charles H. Tegeler

**Affiliations:** ^1^Brain State Technologies LLCScottsdale, AZ, USA; ^2^Department of Neurology, Wake Forest School of MedicineWinston-Salem, NC, USA; ^3^Hypertension and Vascular Research Center, Wake Forest School of MedicineWinston-Salem, NC, USA; ^4^Department of Obstetrics and Gynecology, Wake Forest School of MedicineWinston-Salem, NC, USA

**Keywords:** autonomic nervous system, hemispheric asymmetry, trauma, traumatic brain injury, post-traumatic stress disorder, polyvagal theory, violence, RDoC

## Abstract

A bihemispheric autonomic model (BHAM) may support advanced understanding of traumatic stress effects on physiology and behavior. The model builds on established data showing hemispheric lateralization in management of the autonomic nervous system, and proposes that traumatic stress can produce dominant asymmetry in activity of bilateral homologous brain regions responsible for autonomic management. Rightward and leftward dominant asymmetries are associated with sympathetic high arousal or parasympathetic freeze tendencies, respectively, and return to relative symmetry is associated with improved autonomic regulation. Autonomic auto-calibration for recovery (inverse of Jacksonian dissolution proposed by polyvagal theory) has implications for risk behaviors associated with traumatic life stress. Trauma-induced high arousal may be associated with risk for maladaptive behaviors to attenuate arousal (including abuse of alcohol or sedative-hypnotics). Trauma-induced freeze mode (including callous-unemotional trait) may be associated with low resting heart rate and risk for conduct disorders. The model may explain higher prevalence of leftward hemispheric abnormalities reported in studies of violence. Implications of the BHAM are illustrated through case examples of a military special operations officer with history of traumatic brain injury and post-traumatic stress disorder, and a university student with persisting post-concussion symptoms. Both undertook use of a noninvasive closed-loop neurotechnology – high-resolution, relational, resonance-based, electroencephalic mirroring – with ensuing decrease in hemispheric asymmetry, improvement in heart rate variability, and symptom reduction. Finally, the BHAM aligns with calls for researchers to use brain-behavioral constructs (research domain criteria or RDoC, proposed by the National Institutes of Mental Health) as building blocks for assessment and intervention in mental health science.

## INTRODUCTION AND OVERVIEW

Though studies of the autonomic nervous system (ANS) have historically been dominated by focus on anatomically inferior neural structures or body organs including the heart, gut, skin, and blood vessels, there has also been increasing appreciation for how the ANS is regulated by pathways of the central nervous system that are anatomically and functionally more “upstream” (Saper, 2002; Cechetto and Shoemaker, 2009). The present paper begins on the foundation of multiple studies that have reported lateralization in hemispheric management of arousal or ANS functioning, with the right and left hemispheres being principal managers for the sympathetic and parasympathetic divisions of the ANS, respectively. We propose that the finding of hemispheric laterality in ANS management may be productively integrated with recent thinking that suggests a hierarchical structure in ANS responsivity to stress or trauma (Porges, 2011). The resulting synthesis is a novel ANS model of traumatic stress effects on health and behavior that may have explanatory, predictive, and interventional implications as a new paradigm in trauma studies.

In one way or another, trauma undoubtedly affects the entire brain. To gain initial traction on this complex problem, the bihemispheric autonomic model (BHAM) explains and predicts traumatic stress effects on health and behavior in terms of shifts in hemispheric asymmetry in the activity of bilateral homologous brain regions responsible for autonomic management. Asymmetrical activation in these regions is associated with different forms of physiological arousal and behaviors. Rightward asymmetry in salient regions is associated with acute threats and sympathetic high arousal (fight-or-flight) behaviors. Leftward asymmetry is associated with repeated or severe traumas (or trauma that produces a sense of futility) and a parasympathetic freeze state (physiological and behavioral immobilization). Production of these asymmetries and their associated arousal states is viewed as the expression of relatively autonomous drives for autonomic auto-calibration. That is to say, when the brain “neurocepts” a threatening environment, the ANS will tend to calibrate for high arousal without an individual’s conscious deliberation. Similarly, if the brain neurocepts a stressful or traumatic state that produces no possibility of successful escape (including serial stressors, an overwhelming stressor, or emotional abandonment), then the ANS will tend to calibrate for a freeze response, again without the subject’s thinking or willing.

Autonomic auto-calibration, demonstrable at the level of cerebral hemispheres, may occur in response to trauma, but also as processes or behaviors to support recovery (or attempted recovery) from trauma. Drives for autonomic auto-calibration out of traumatic stress states may explain some behavioral repertoires associated with traumatic life experience, and this aspect of the BHAM is developed in alignment with the hierarchical structure of ANS behavioral responsivity proposed by polyvagal theory (Porges, 2011). Polyvagal theory posits that vagal inhibition of sympathetic arousal, in safe contexts, is the highest-order mode of ANS operation, followed by sympathetic high arousal for acute threats, and finally parasympathetic freeze mode for persistent or severe trauma, as mode of last resort. An inverse view of this hierarchy – in conjunction with postulated existence of autonomous drives for recovery – suggests that individuals in parasympathetic freeze mode may be at risk for generating compensatory high-arousal behaviors to depart the freeze mode, including conduct disorders and substance abuse, to generate greater experience of subjective *feeling*. Individuals in sympathetic high-arousal mode may be at risk for arousal-attenuating behaviors including substance abuse or medication dependence or abuse. Evidence from criminology appears to support the BHAM explanation for history of traumatic life events and high-arousal behavioral risk, in that individuals with propensity for violence have increased prevalence of leftward hemispheric asymmetry or left-hemispheric aberrancy.

The paper illustrates the model through two case examples of individuals with histories of traumatic stress including mild traumatic brain injury, in which reduction of asymmetrical activity in brain regions associated with autonomic management appeared to reflect improved upstream regulation in the ANS. Comment on these cases includes suggestion that the BHAM is in alignment with calls from the National Institutes of Mental Health (NIMH; Cuthbert and Insel, 2013) for researchers to focus on biologically valid brain-behavioral constructs, rather than symptom checklists and population statistical criteria, to make meaningful progress in mental health science. The discussion concludes by proposing that recently proposed hierarchical understanding of ANS responsivity integrated with appreciation of hemispheric lateralization in ANS management, may support a new scientific paradigm (Kuhn, 1962) for research and intervention related to the ANS with broad implications for health and behavior.

## STUDIES REPORTING HEMISPHERIC ASYMMETRY IN MANAGEMENT OF AROUSAL OR AUTONOMIC FUNCTIONING

Many studies have reported hemispheric asymmetry in management of arousal levels, either demonstrating or potentially implying a distinct form of ANS activity associated with each hemisphere. We review representative studies in four categories: consequences of unilateral brain dysfunctionality on measures of arousal, studies involving direct stimulation or inactivation of brain structures, psychophysiological studies in healthy subjects, and studies related to stress effects on hemispheric or neurocardiac functioning. Several of the studies have converged on the bilateral insular cortices as being key sites for lateralization in hemispheric management of the ANS, and implications of this finding are addressed in the case study section. For purposes of this literature review we highlight hemispheric laterality as the common unit of analysis.

Gainotti (1972) compared 80 patients with left-sided lesions to 80 patients with right-sided lesions, with respect to their behaviors during neuropsychological exams. He found that those with left-sided lesions were more likely to demonstrate emotional tendencies for “catastrophic reactions” (anxiety, tears, swearing), and those with right-sided lesions were more likely to show “indifference reactions” (indifference, minimization, or anosognosia). Other teams (Heilman et al., 1978; Morrow et al., 1981) extended his findings with psychophysiological measures showing decreased galvanic skin resistance with a stimulus, indicative of hypoarousal, in patients with right-hemispheric lesions, compared to patients with left-hemispheric lesions or controls without brain injury. The above authors largely refrained from positing a causal role of the left hemisphere to explain findings in patients with right-sided lesions, proposing instead injury to thalamo-cortical loops (Heilman et al., 1978) or disruption of right hemispheric functionality for emotional processing (Gainotti, 1972; Morrow et al., 1981). More recently, Daniele et al. (2002) reviewed electrocardiograms of 352 hospitalized individuals with completed ischemic strokes and found that those with right-hemispheric injuries were more likely to have cardiac arrhythmias than those with left-sided injuries. They postulated that right-sided injuries were more likely to disinhibit right-sided neural mechanisms for sympathetic regulation of heart rate. Finally, Avnon et al. (2004) reported that in a group with unilateral migraine, those with left-sided headache were more likely to have augmented parasympathetic responses – vasodilatation and bradycardia in response to a mild stressor (soapy water to the eye) – than were those with right-sided symptoms. In contrast to the earlier studies, this report suggested a direct role of the left hemisphere in producing parasympathetic responses.

Peri or intra-operative epilepsy surgeries have allowed more direct inferences about the causal role of brain structures. Zamrini et al. (1990) investigated cardiovascular responses from unilateral hemispheric inactivation by intra-carotid amobarbitol, in 25 epileptics undergoing preoperative evaluation for epilepsy surgery. Heart rate increased after left hemisphere inactivation, and decreased after right hemisphere inactivation. Spurred by interest in possible mechanisms of cerebrogenic sudden death in epileptics, Oppenheimer et al. (1992) performed intra-operative stimulation of the insular cortex prior to temporal lobectomy for seizure control. They found that stimulation of the left insula (in comparison to the right) produced more bradycardia and blood pressure depressor responses than tachycardia and pressor effects. The opposite was true of right insula stimulation. Other studies (also based on unilateral intra-carotid amobarbitol infusion in epileptics) corroborated opposing hemispheric roles for autonomic cardiovascular control using spectral analysis of heart rate (Yoon et al., 1997) and measures of blood pressure and baroreflex sensitivity (Hilz et al., 2001). Collectively, these studies lend stronger evidence that neural mechanisms in the left hemisphere have an independent, efferent, and parasympathetic role in autonomic modulation.

Evidence that hemispheric lateralization of autonomic management exists for human subjects without neurological disease has been provided through two sets of experiments by Wittling (1990) and Wittling et al. (1998a,b). In the first study, Wittling (1990) and Wittling et al. (1998a,b) showed a romantic film to 50 young adults through a technique of stimulus presentation to a single hemisphere at a time. Right hemispheric film presentations caused significantly greater increases to blood pressure, especially for the female subjects. In the subsequent study, they used the same single hemisphere stimulation technique to show two different films (to control for the effects of emotionality as such, independent of lateralized presentation effects) to 58 young adults. One film was emotionally challenging (“Schindler’s List,” a film about the pogrom of Jews in Germany), while the second was a scenic film of peaceful pictures. Right hemisphere stimulation was found to be associated with increased ventricular myocardial activity, and left hemispheric stimulation was found to be associated with higher values for the high-frequency spectral component of heart rate variability, a commonly used metric for parasympathetic activation. These studies have represented an important step towards a BHAM by further showing independent roles of the hemispheres, and in subjects without brain injury.

The fourth category of studies supporting a two-hemisphere view of ANS management includes reports that relate to the topic of stress effects on hemispheric or neurocardiac functioning. Rabe et al. (2006) compared electroencephalographic activity of 22 individuals with post-traumatic stress disorder (PTSD) due to motor vehicle accident, 21 individuals who had been through a motor vehicle accident but without PTSD, and 23 healthy controls. Those with PTSD showed greater right-hemispheric activation than the other groups (decreased alpha band on the right compared to the left), when exposed to trauma-related material, and the degree of their asymmetry was correlated with PTSD symptom severity. The same researchers went on to show that rightward asymmetries tended to be reduced after a successful cognitive-behavioral therapy intervention (Rabe et al., 2008). Right-temporal lobe activation has also been reported in a magneto-encephalography study of PTSD (Engdahl et al., 2010), though no inference was made by the authors to associate asymmetry with autonomic mechanisms. In an acute stressor paradigm (performance of rapid calculations), Gray et al. (2007) studied scalp-derived electrical potentials associated with cardiac function, in ten men with heart disease. They found that negativity of a heart-evoked potential (HEP, an electrical signal from the scalp, synchronized with the heart beat) at left temporal and left lateral frontal regions was correlated with changes in cardiac output and cardiac repolarization homogeneity. While this report appears to add to the case for a bihemispheric model of autonomic management, perhaps even more significantly it showed that cortical signals related to autonomic cardiac regulation could be detected through noninvasive measures at the scalp. And though the authors approached the study as a means to better understand afferent cardiac signaling to the cortex, they were careful to recognize that the cortical potential they measured could instead be representing efferent signaling from the cortex to control the heart rate. Finally, recently we have reported (Tegeler et al., 2013) in a heterogeneous cohort of individuals with stress-associated conditions, that leftward asymmetry in temporal lobe high-frequency electrical activity measured from the scalp is negatively correlated with heart rate and positively with heart rate variability.

It should be noted that not all learned opinion is in agreement regarding the defensibility of a consistent model of lateralized cortical cardiovascular sympathetic and parasympathetic representation. One general refutation of this hypothesis has been presented in the discussion of a retrospective study of individuals hospitalized for video-EEG monitoring whose medical records included evidence of bradycardia or bradycardia-related clinical events (Britton et al., 2006). The authors identified 13 cases that met their inclusion criteria, out of 6168 who underwent video-EEG monitoring over a 14-year period, and they found no consistent hemispheric lateralization of seizure activity in these patients. This study by Britton et al. (2006) is a helpful counterpoint, though it bears mentioning that lack of evidence for correlation between an autonomic effect and a specifically hypothesized form of asymmetry (e.g. lateralized seizure activity) permits a weaker type of inference – only that the autonomic effect being studied was not associated with that specific form of asymmetry – and does not constitute evidence that lateralization in autonomic management does not exist.

## THE BIHEMISPHERIC AUTONOMIC MODEL

The studies reviewed in the preceding section collectively suggest that an accurate and comprehensive view of autonomic functioning requires consideration of the independent roles of the left and right hemispheres, and especially patterns of asymmetrical hemispheric activation in brain regions responsible for autonomic management. In this section, we integrate the above data into a BHAM that encompasses the above findings while also proposing a way to understand the dynamics of trauma effects on the brain and brain influences on behavior. We propose the existence of relatively autonomous drives for autonomic auto-calibration that may find expression as shifting forms and degrees of asymmetrical hemispheric activation. For purposes of this paper, the model is deliberately qualitative and conceptual. We hope the model will encourage empirically precise, quantitative, and hypothesis-driven studies, even as we hope it facilitates additional conceptual and theoretical innovations across health and behavioral sciences.

### FOUR THESES OF THE MODEL

Thesis One. *Relative symmetry in activation of bilateral cerebral hemispheric regions responsible for management of the autonomic nervous system is likely to be associated with an organismal state of relative autonomic optimality, characterized by relatively small and healthy fluctuations between leftward and rightward asymmetry of activity in those regions*. This state of relative symmetry, fluctuating towards either leftward or rightward asymmetry but not to extreme degrees or for prolonged lengths of time, corresponds to a relative capacity for healthy and adaptive fluctuation. In a complex and changing environment, it is healthy, needful, and subjectively enjoyable, to move smoothly and fluidly between parasympathetic states of rest and calm, and sympathetic states of increased arousal, action, and overt excitement.

Thesis Two. *Rightward dominant asymmetry may arise in hemispheric regions responsible for management of the autonomic nervous system, in association with traumatic experience or perception of threat, producing a tendency for high arousal physiology that is likely to be maladaptive in its persistence*. The right hemisphere-dominant autonomic state may be adaptive, for example in a military serviceperson in armed combat, or in an individual living in close quarters with someone who behaves abusively. However, the persistence of such asymmetry and associated high-arousal physiology is likely to be maladaptive for non-threatening contexts. The same soldier returning from war will predictably have difficulties in maintaining the calm and restful parasympathetic state needful for civilian life. Similarly, the individual under threat of abuse in their home may feel maladaptively aroused when they go to the workplace or attempt to enjoy low-risk social encounters. Individuals in this state may be at risk for compensatory behaviors to attenuate the high arousal associated with right-dominant autonomic asymmetry, for example substance abuse or medication dependence or abuse.

Thesis Three. *Leftward dominant asymmetry may arise in hemispheric regions responsible for management of the autonomic nervous system, especially in association with severe trauma or perception of futility, producing a risk for compounded maladaptations – tendencies both for persistent immobilization physiology, as well as compensatory high arousal behaviors*. A leftward dominant state may be characteristic of an individual faced with stress or trauma that overwhelms the utility of high arousal (for fight or flight) or otherwise presents no exit options, thereby inducing a state of behavioral freeze or shutdown. For example, leftward asymmetry might characterize a soldier who has had severe traumatic stress and is now emotionally numb (frozen), or an individual who feels abandoned by a care-giver or loved one. Such a state may be adaptive as a way to withdraw from complex environments (and decrease risk of further injury), but if persisting it will prevent healthful engagement with life by most any reasonable measure. Paradoxically, high-arousal behaviors may also be expressed by those with immobilization physiology as compensatory processes (to depart the freeze state). Risk for producing these behaviors, which may include conduct disorders, rage, or substance abuse, is predicted to represent a significant burden of suffering for those in the parasympathetic freeze state (see Autonomic Auto-calibration May Explain Increased Risk for High-Arousal Behavior in Individuals who are in a Parasympathetic Freeze State).

Thesis Four. *Movement between the states of symmetry and asymmetry described in Theses One through Three is a function of two main influences – relatively autonomous drives for autonomic auto-calibration, and higher-order self-regulatory processes managed by the prefrontal cortex.* Exposure to stressors or trauma will be associated with relatively autonomous drives for autonomic auto-calibration from relative symmetry to rightward dominance, and/or leftward dominance, whereas processes for recovery will tend to be associated with auto-calibration from leftward or rightward dominance, toward relative symmetry. Autonomous drives for autonomic auto-calibration may be guided by conscious choice-making and other capacities associated with the executive role of the prefrontal cortex, that represent a rudder and decision-making apparatus for navigation and management of environmental contexts and neural energetics that together produce asymmetries.

The distinction made in the model between stresses that produce rightward or leftward asymmetry owes much to polyvagal theory (Porges, 2011), which has reconceptualized autonomic responsivity in hierarchical terms, distinguishing between self-calming (typically healthful) and immobilizing (trauma-induced and typically maladaptive when persisting) functionalities of the vagus nerve. Roughly, the first three theses of the BHAM may be considered to correspond to functionality of the myelinated (“smart” or “social”) vagus, the sympathetic division, and the unmyelinated (“vegetative”) vagus, respectively. Polyvagal theory is discussed further in Autonomic Auto-calibration May Explain Increased Risk for High-Arousal Behavior in Individuals who are in a Parasympathetic Freeze State.

**FIGURE 1:**
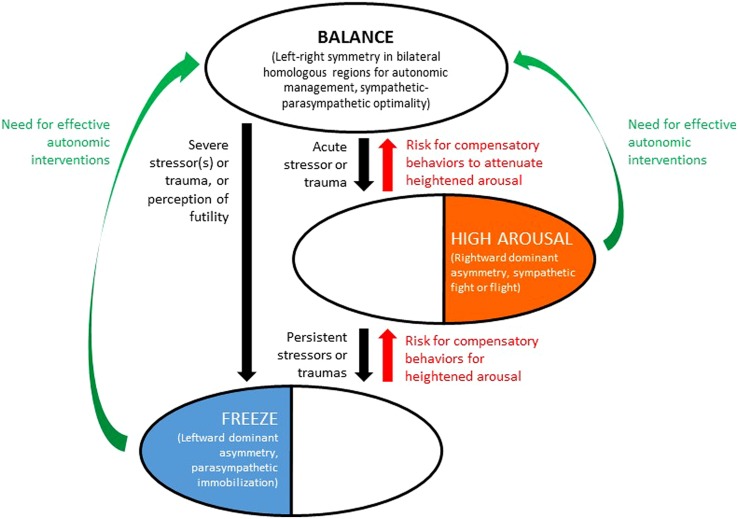
**Bihemispheric autonomic model (BHAM) for traumatic stress effects on health and behavior through auto-calibration of arousal**. Top oval denotes state of relative symmetry (balance) in activity of bilateral homologous brain regions responsible for autonomic management. Middle oval denotes rightward dominant asymmetry in activation of the same brain regions, indicative of traumatic stress associated with high arousal. Bottom oval denotes leftward asymmetry in activation of the same regions, indicative of traumatic stress associated with parasympathetic freeze mode. Recovery from trauma may be associated with compensatory adverse behaviors (including but not limited to conduct disorders, especially from freeze state to progress to high arousal; and varying forms of substance abuse or medication dependence, to achieve state of balance). Effective autonomic interventions are needed, that can support recovery of balance and decrease likelihood of compensatory adverse behaviors.

We reiterate that the BHAM as presented in this paper is qualitative and conceptual. It is intended to explain and predict tendencies and risks, not causality, and in its current form is a deliberate simplification. Many individuals in a right-hemispheric autonomic state (Thesis Two) may be “driven high-achievers” without pathological high arousal. Similarly many individuals in a left-hemispheric autonomic state (Thesis Three) may have relatively withdrawn personalities that are not necessarily dysfunctional. Individuals may also have mixed forms of trauma history (producing both sympathetic high arousal and parasympathetic freeze tendencies) associated with mixed forms of right/left overactivation that masks the underlying hemispheric dynamics. With these cautions and caveats, the main elements of the BHAM theses are illustrated in **Figure 1**.

Notably, Craig (2005) has proposed a model of forebrain emotional management that similarly depends on lateralization of hemispheric management of the ANS, and in a broad sense his conceptualization overlaps with Thesis One. The Craig model helpfully articulates the roles of the right and left hemispheres for homeostatic opponent processes, permitting catabolic and anabolic energy management through the sympathetic and parasympathetic divisions, respectively. In contrast to the BHAM, the Craig model tends to focus on afferent over efferent pathways for the brain’s interaction with the peripheral ANS and views the parasympathetic role of the left hemisphere primarily in terms of its role for self-calming and interoception. Thesis Two of the model overlaps with discussion from Rabe et al. (2006), who also propose a model correlating rightward hemispheric asymmetry with increased arousal. Davis et al. (2013) recently showed that individuals with post-traumatic stress disorder and comorbid substance use disorder had decreased startle responses compared to those with post-traumatic stress disorder alone (self-medication hypothesis), lending support to the idea that autonomic auto-calibration to recover from high arousal may be a driver for adverse compensatory behaviors. We are not aware of other writings that advance the ideas contained in Theses Three or Four.

### EXPLANATORY VALUE OF THE MODEL

#### Model subsumes earlier findings related to hemispheric lateralization of arousal or autonomic management

The BHAM subsumes the reports cited in Section “Studies Reporting Hemispheric Asymmetry in Management of Arousal or Autonomic Functioning.” Gainotti’s (1972) early finding that individuals with right-sided lesions were more likely than those with left-sided lesions to have “indifference” reactions, can be re-interpreted to implicate efferent left-hemispheric influences for freeze behaviors – avoidance or lack of engagement – disinhibited by the right-sided injury. Similar re-interpretations can be offered for the findings of Heilman et al. (1978) and Morrow et al. (1981). The findings of Avnon et al. (2004) also align with the model, suggesting that left-sided migraine carries a burden of greater parasympathetic shutdown physiology – in the form of bradycardia and vasodilatation – after a stressor, perhaps due to enhanced excitably of left-hemispheric circuits. Bradycardia or vascular depressor responses reported by several of the other studies (Zamrini et al., 1990; Oppenheimer et al., 1992; Hilz et al., 2001; Tegeler et al., 2013) are also consistent with the idea that the left hemisphere mediates a parasympathetic freeze response.

#### Autonomic auto-calibration may explain increased risk for high-arousal behavior in individuals who are in a parasympathetic freeze state

In this section, we further develop the explanation of behavioral risks associated with a history of trauma exposure (Thesis Three). Fuller explanation of this idea is supported by review of the polyvagal theory. The polyvagal theory proposes that the vagus nerve includes branches that are anatomically and functionally distinct, deriving from different periods of vertebrate evolutionary phylogeny. A myelinated branch is associated with fine-tuned self-calming, social communication skills and physiological regulation, and inhibition of sympathetic arousal in environments perceived to be safe. An unmyelinated branch is associated with neurogenic bradycardia and other freeze-mode behaviors in the presence of severe stressors, when movement may appear to be futile or dangerous, or in the setting of novel stimuli. In traumatic context, the freeze-mode produces hypoarousal, subjective emotional numbness, avoidant behaviors and social disengagement, and in severe form dissociation.

Polyvagal theory conceives autonomic regulation in hierarchical terms, in contrast to dominant models that view sympathetic and parasympathetic functions as opponent processes (or as being in paired antagonism). The myelinated vagus is normatively “on top,” functional in safe and restful environments and inhibitory of sympathetic mobilization. In the setting of an acute threat that requires mobilization or otherwise overwhelms the capacity for self-calming, sympathetic arousal is “second in command” and can provide fight or flight responses. If sympathetic mobilization behaviors are inadequate for the stressor or otherwise not adaptive for the presented need, then the unmyelinated vagus may be engaged as the mode of last resort, producing freeze or shutdown behaviors and physiology. These relationships are explained to be consistent with the Jacksonian principle of dissolution wherein evolutionarily more primitive structures rise to dominance when the higher are rendered inadequate.

The existence of *relatively autonomous drives for autonomic auto-calibration* has been proposed in our model as a way to describe dynamics of movement between different autonomic states. This postulate overlaps with polyvagal theory’s incorporation of the concept of Jacksonian dissolution. Shifts to sympathetic high arousal or parasympathetic freeze states associated with stress exposures may represent needful, automatic, and calibrated responses to perceived threats. Whereas Jacksonian dissolution explains trauma effects to produce relatively more primitive functioning, the concept of autonomic auto-calibration can also be used to explain behaviors associated with *recovery* (or attempted recovery) from stress or trauma in the direction of reconstitution or greater order. The ubiquity of relaxation strategies (from formal introspective meditation habits to alcohol or sedative-hypnotic use), can be understood as the response to the need of stressed or traumatized individuals to inhibit sympathetic arousal (Thesis Two).

The adverse effects of excess sympathetic arousal – and medical or self-directed strategies to dampen arousal – are commonly recognized by both health professionals and much of popular culture. In contrast the parasympathetic freeze state is less well known, less obviously amenable to intervention, and perhaps even more problematic as a health risk. In extreme cases, neurogenic bradycardia can lead to cardiac arrest (including fetal demise in obstetric contexts), and it may be a mechanism for cerebrogenic sudden death in epileptics. Unmyelinated vagal freeze-mode mechanisms likely contribute to asthma (Porges, 2011). Furthermore, a persisting freeze state may be associated with burdens of suffering independent of the risks of acute cardiac or respiratory shutdown, and also independent of the psychosocial disturbances or discontinuities that accompany emotional numbness or avoidant behaviors as such.

In explication of Thesis Three, we propose that an important burden of suffering associated with the freeze mode is related to autonomic auto-calibration in the direction of recovery from trauma. The hierarchical logic of polyvagal theory appears to suggest that recovery from a traumatic freeze state that may be associated with disproportionate engagement of the unmyelinated vagus requires some degree of transition through a sympathetic high-arousal state, before one can regain (or gain) the self-calmed state of the myelinated vagus. *Autonomic auto-calibration to depart a parasympathetic freeze state may be expressed as a drive for movement towards high-arousal states, which may be socially dysfunctional but nonetheless experienced as a “step-up” in the hierarchy of autonomic states described by polyvagal theory.* It is established for example that a history of early life abuse or neglect confers a risk of anti-social behaviors or conduct disorder (Weiler and Widom, 1996; Maniglio, 2014). Autonomic auto-calibration may explain this risk as the expression of relatively autonomous drives, in individuals who are in a parasympathetic freeze state, to engage in behaviors associated with heightened arousal. Broadly, we hypothesize that heightened-arousal behaviors driven by a need to depart a parasympathetic freeze state may take a variety of forms, from conduct disorders including rage, to drug abuse (especially, but not only, stimulants) and possibly suicidality.

Autonomic auto-calibration in relation to the freeze state would appear to have specific salience for research on the psychological trait called “callous-unemotional.” Callous-unemotional traits have been found greater in children with experience of trauma, with the relationship being mediated by self-reported numbness to emotions (Kerig et al., 2012). In turn, a higher degree of the callous-unemotional trait in early adolescence has been shown to be a strong predictor of anti-social outcomes during adolescence and adulthood, including delinquency and arrests (McMahon et al., 2010). Callous-unemotional traits may be a function of traumatic stress that produces the parasympathetic freeze state, and the resulting greater risk for conduct disorders may arise from a drive to be unfrozen, to experience *feeling*.

Corroborative evidence for the physiological dimension of Thesis Three appears to exist in the association between low resting heart rate in children and adolescents, and anti-sociality. In reviews spanning nearly twenty years, the criminologist Adrian Raine has concluded that low resting heart rate is the best-replicated biological correlate of anti-sociality in children and adolescents (Raine, 1996, 2002; Glenn and Raine, 2014). Low resting heart rate correlates with anti-sociality independently of multiple other risk factors, and the significance of the relationship has been confirmed in prospective studies. Raine has invoked both psychological and neural functional explanations for this relationship, and his reference to “stimulation-seeking theory” is of special pertinence to the BHAM being proposed in this paper. Low resting heart rate may represent a subjectively unpleasant low-arousal state that encourages behavioral processes including anti-sociality to mitigate or depart the experience of that state. This explanation is essentially identical with the concept of auto-calibration of arousal to depart parasympathetic freeze mode. Intriguingly, Raine (2002) has further noted that the finding of low resting heart rate (increased parasympathetic efferent control) seems to be at odds with other findings showing that anti-social children have decreased vagal tone (decreased respiratory sinus arrhythmia or heart rate variability, indicative of decreased parasympathetic efferent modulation). However, this paradox is entirely consistent with the existence of two distinct forms of vagal functionality proposed by polyvagal theory. As to the cause for low resting heart rate, the construct of auto-calibration of autonomic arousal encompasses early life traumatic exposures and thus may explain developmental aspects of anti-sociality more robustly than can explanations based on strictly genetic heritability.

The challenge of the freeze state and the relatively autonomous drive for autonomic auto-calibration is perhaps most sharply illustrated by the plight of many US veterans who have returned from the wars in Iraq and Afghanistan. Many or most of these servicemen and women will have experienced significant degrees of stress or trauma. Depending on individual factors, their autonomic physiology is likely to be characterized by significant sympathetic mobilization tendencies, but also marked degrees of parasympathetic freeze mode. Some expressions of high arousal may reflect intrinsic elevation in sympathetic activity, while others may represent a drive to disengage from the freeze state. The latter expressions might include propensity for violence, substance abuse, and suicidality – all of which are highly prevalent in these veterans (Institute of Medicine, 2013).

#### The bihemispheric autonomic model may explain left-hemispheric asymmetries or abnormalities associated with violence

A recurrent question in criminology is whether or to what degree biological factors may influence propensity for criminal behavior. This topic has been pursued intermittently over the twentieth century with respect to cerebral asymmetry, unilateral hemispheric dysfunctions, and laterality preferences (Nachson and Denno, 1987). One finding that emerged from early work is that left hemispheric EEG abnormalities, especially in the temporal lobe, tend to be more frequent in violent compared to non-violent subjects, and more recent studies have corroborated that finding. Using positron emission tomography, Volkow and Tancredi (1987) reported left temporal lobe metabolic abnormalities in four psychiatric patients with a history of repetitive, purposeless, violent behavior. Convit et al. (1991) recorded EEG’s of 21 violent male psychiatric inpatients and found that increased leftward fronto-temporal and temporal asymmetry in the low frequency range (delta band) was correlated with increased violence. Wong et al. (1994) studied 372 subjects in a maximum security mental hospital and reported that those categorized to be in the highest tertile for violence had a markedly higher number of temporal lobe EEG abnormalities (without mentioning whether asymmetries were present). Pillman et al. (1999) reported that, among 222 defendants in a state court who were seen for pretrial psychiatric assessment, the ten individuals with focal left temporal lobe EEG abnormalities had a significantly higher number of violent offenses, than those without abnormalities. More recently, leftward temporal lobe EEG slowing and diminished arousal to emotional stimuli were reported in a case study of a serial killer (Ostrosky-Solis et al., 2008), and leftward temporal lobe high-frequency brain electrical asymmetry was observed in a group of five violent inmates in a medium-security prison (Gerdes, unpublished observations). In some of these studies, left-sided electrical abnormalities or leftward asymmetries have been interpreted to indicate deficits in language or cognitive processing skills that led the individuals to rely on violence as instrumental means for social interaction.

It should be pointed out that the above studies include reports of both left-sided abnormalities of EEG and leftward asymmetry of electrical activity. While equivalence should not be presumed between abnormalities and asymmetries, nonetheless on a preliminary basis we interpret these studies to suggest that, as with the patients with left-sided migraine symptoms (Avnon et al., 2004), that aberrant or excess left-sided electrical activity represented in these subjects was an indicator of augmented parasympathetic freeze physiology. Review of these data from the perspective of the BHAM, including its concept of autonomic auto-calibration from freeze state, suggests an explanation for the violence of individuals with left-sided defects or excess in brain electrical activity that is independent of the left hemisphere role for language or analytic cognition. Individuals prone to violent behavior may disproportionately have a history of severe life stress adequate to promote the parasympathetic freeze state, associated with leftward asymmetry in the activity of brain regions responsible for autonomic management. A relatively autonomous drive for autonomic auto-calibration may have been expressed, in the individuals in these studies, as propensity for violence.

The BHAM interpretation that risk for violence associated with leftward asymmetry or left-sided abnormality does not contradict the idea that cognitive or language deficits associated with left-sided aberrancy contribute to violent tendency. The combined influence of these factors could easily be greater than either on its own. Furthermore, study design and statistical aspects of the above findings permit a conclusion no stronger than to suggest that leftward dominant asymmetry merits ongoing investigation as a possible relative risk factor for violence, not a determinant of violence. Even if leftward dominant asymmetry is validated as a risk factor for conduct disorder or violence, we hypothesize that only a minority of individuals with leftward dominance will manifest flagrant behavioral disturbance. We reiterate the role of the prefrontal cortex for supporting an individual to steer behaviors and navigate environments, whatever one’s prevailing asymmetry. With those caveats, we propose that the BHAM, including the concept of autonomic auto-calibration, may explain previously unexplained portions of the likelihood to enact dysregulated behaviors, among individuals with a history of severe traumatic stress. The model also has implications for intervention, and these are explored in the following section.

## ILLUSTRATIONS OF THE BIHEMISPHERIC AUTONOMIC MODEL INCLUDING IMPLICATIONS FOR INTERVENTION

### A PARADIGM OF INTERVENTIONAL RESEARCH BASED ON THE BIHEMISPHERIC AUTONOMIC MODEL

The two case examples below are drawn from participants enrolled in an IRB-approved, open label, feasibility study at Wake Forest School of Medicine, exploring use of a noninvasive computer-guided neurotechnology for individuals with a variety of conditions, many associated with stress or psychophysiological dysregulation. The technology is called high-resolution, relational, resonance-based electroencephalic mirroring (HIRREM^®^, or Brainwave Optimization^®^), and it is a non-medical device designed to facilitate relaxation and auto-calibration of neural oscillations (Gerdes et al., 2013). The technology produces closed-loop acoustic stimulation (audible tones of variable pitch and timing) such that the brain tends to self-optimize its electrical activity patterns, shifting towards greater symmetry between left and right hemispheres, and more optimized ratios of energy along the brain electrical frequency spectrum. For these subjects, the technology was provided as a series of sessions, typically 90–120 min duration (and up to two sessions per day), with each session composed of a series of 4–9 protocols (6–40 min per protocol), conducted predominantly with eyes closed while at rest. Protocols target multiple brain regions including temporal, frontal, frontal pole, central, parietal, occipital, cerebellar, and occipital lobes and locations. The technology is a “whole-brain” approach, but for purposes of explicating the BHAM, attention is paid in the case illustrations to temporal lobe activity only. As reviewed above, surface readings of brain electrical activity from temporal locations have been found to correlate with peripheral measures of autonomic cardiac control (Gray et al., 2007; Tegeler et al., 2013), and temporal scalp locations have specifically been proposed to be sites for recording or influencing afferent or efferent autonomic activity in the insular cortex (Gray et al., 2007; Montenegro et al., 2011; Gerdes et al., 2013; Okano et al., 2013).

Participants completed a variety of measures at baseline and again after completing the sessions. Self-reported symptom inventories included the Insomnia Severity Index (ISI), a 7-item survey that assesses the severity, nature, and impact of insomnia symptoms on quality of life over the previous two weeks, with possible scores ranging from 0 to 28 (Morin et al., 1993). Scores of ≥8 suggest clinically relevant symptoms of insomnia, and a change of seven points reflects a meaningful change. The Center for Epidemiologic Studies Depression Scale (CES-D) is a 20-item survey, with possible scores from 0 to 60, that assesses affective depressive symptomatology to screen for risk of depression (Radloff, 1977). Scores of ≥16 suggest clinically relevant symptoms of depression, and a change of eight points reflects a meaningful change. The Post-traumatic stress disorder (PTSD) Checklist-Civilian Version (PCL-C), is a 17-item inventory to evaluate multiple symptoms of post-traumatic stress, with scores ranging from 17 to 85 (Weathers et al., 1993). Scores of ≥44 points suggest clinically relevant symptoms of PTSD for the PCL-C, and a change of 16 points reflects a meaningful change.

Baseline data collection also included continuous recordings of blood pressure and heart rate (bpm) data, acquired from noninvasive finger arterial pressure measurements, for a minimum of 5 min while subjects were in the supine position. Systolic blood pressure and RR interval data acquired (BIOPAC acquisition software, Santa Barbara, CA, USA) at 1000 Hz were analyzed using Nevrokard BRS software (Nevrokard BRS, Medistar, Ljubljana, Slovenia) to produce measures of heart rate variability, reported here as the standard deviation of the normal beat to beat interval (SDNN, ms), and baroreflex sensitivity (BRS, ms/mmHg), which we report according to the sequence method.

Each participant had a baseline assessment to obtain information regarding electrical frequencies and amplitudes (Gerdes et al., 2013). The assessment included 3 min recordings obtained from at least six standard locations on the scalp (using placements from the 10–20 system, F3/F4, C3/C4, P3/P4, T3/T4, FZ/OZ, and O1/O2, 1 min each for eyes closed, partially closed, and eyes open), with the participant at rest (eyes closed, partially closed) and while carrying out a cognitive task (eyes open). The assessment is intended to provide a “snapshot” of relative symmetry between homologous brain regions, as well as the distribution of amplitudes among different frequency bands at each location. Data from the assessment were used to identify the protocols for the first intervention session, and data from each intervention session were used to guide protocol selections for subsequent sessions. For purposes of the following case illustrations, spectrographs of 1 min averages of amplitudes at bilateral temporal lobes, eyes closed, are shown for the assessment and the penultimate minute of the temporal lobe protocol for the penultimate HIRREM session.

### CASE 1: LEFTWARD TEMPORAL LOBE BRAIN ELECTRICAL ASYMMETRY IN A SPECIAL OPERATIONS MILITARY OFFICER WITH MILD TRAUMATIC BRAIN INJURY AND POST-TRAUMATIC STRESS DISORDER

A 29-year-old man, deployed as part of a US military special operations unit, experienced a mild traumatic brain injury (mTBI) when he was in close proximity to an exploding rocket-propelled grenade. He was diagnosed with mTBI and post-traumatic stress disorder. He had been in good overall health prior to the mTBI, with the exception of several years of insomnia, requiring medications. He reported that his primary symptoms were severe insomnia, headaches, and impaired memory, both short and long term. The traumatic event occurred 15 months prior to enrollment. During the period between the mTBI and enrollment, he reported having tried numerous treatments including both medical therapies and non-traditional approaches (cognitive processing therapy; prolonged exposure; group therapy; anti-depressant medication; adrenal optimization; dietary changes; fitness changes; nerve blocks; nerve ablations; acupuncture; transcranial magnetic stimulation; meditation; massage therapy; pain medication; ketamine infusions; bio-feedback; sleep medications; service dog) from which results had been by his estimation “mixed and relatively limited.” At the time of enrollment, pertinent medications included eszopiclone (3 mg nightly), melatonin (30 mg nightly), venlafaxine XR (225 mg daily), and thyroid hormone (2 grains daily).

**FIGURE 2:**
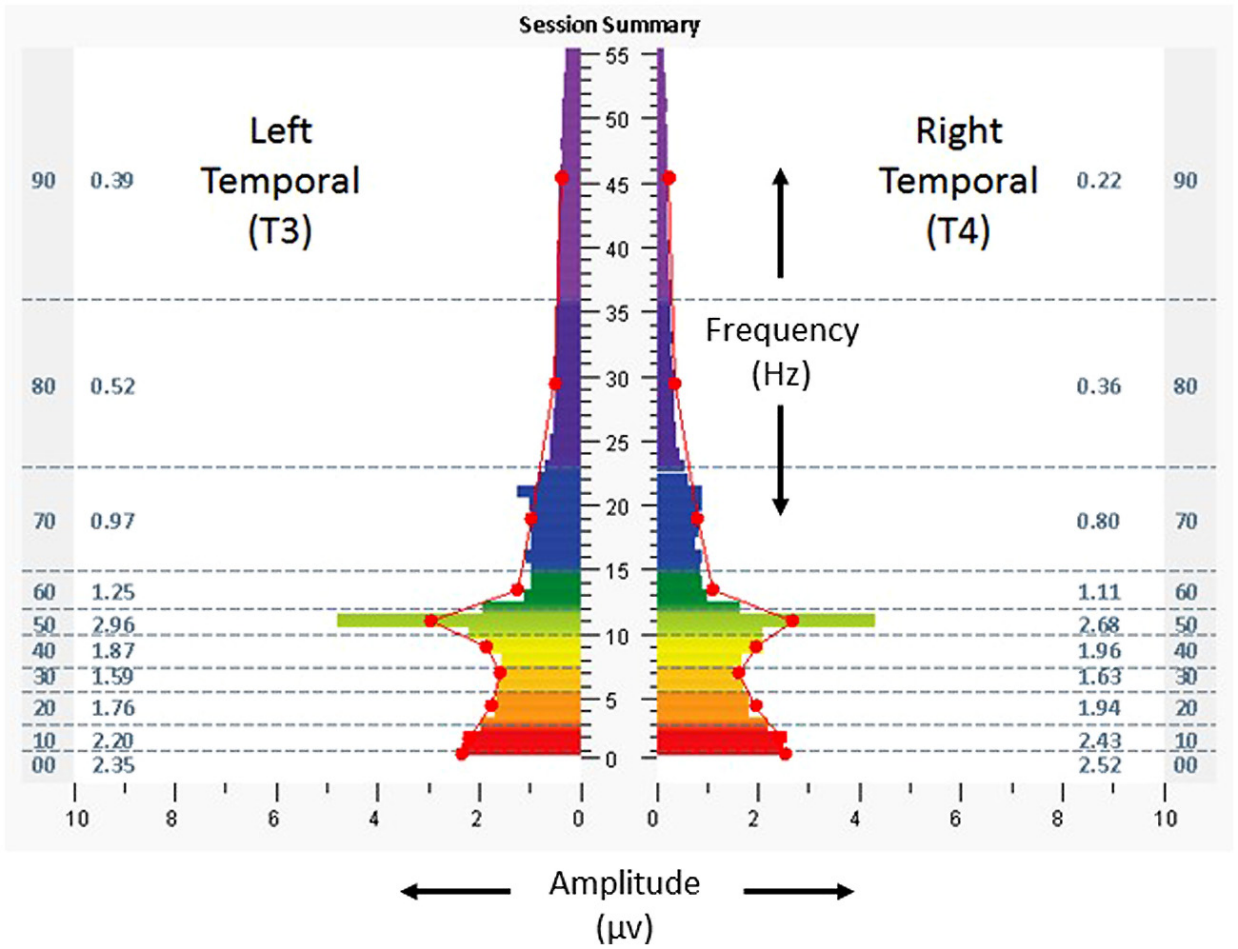
**Spectrographs of left and right temporal lobe brain electrical activity for 29-year-old US military special operations officer with history of mTBI and PTSD (Case 1) at baseline, before undergoing neurotechnology intervention for auto-calibration of neural oscillations**. Data were collected from T3 and T4 in 10–20 system, with frequency (Hertz, Hz, vertical axis), plotted against amplitude (microvolts, µv, horizontal axis). Individual color bars reflect amplitude averages for one minute of recording, eyes closed, at rest, without stimulation. Columns to the left and right of the color bars denote ten frequency bands of aggregated data (00: < 1.0 Hz; 10: 1.0–3.0 Hz; 20: 3.0–5.5 Hz; 30: 5.5–7.5 Hz; 40: 7.5–10.0 Hz; 50: 10.0–12.0 Hz; 60: 12.0–15.0 Hz; 70: 15.0–23.0 Hz; 80: 23.0–36.0 Hz; 90: 36.0–48.0 Hz) and numerical values for averages in those ranges.

On baseline assessment, at homologous temporal lobe regions (T3/T4) with eyes closed (**Figure 2**), there was a leftward (T3) dominant pattern in the higher frequencies (amplitudes 20–74% greater than the right, at T4). Scores for the ISI, CES-D, and PCL-C were 28, 34, and 78, respectively. Resting heart rate, SDNN, and BRS were 53 bpm, 65 ms, and19.2 ms/mmHg, respectively. The subject received a total of 22 HIRREM sessions over 12 days, during which time he self-tapered and/or discontinued his various medications. He reported no adverse events in association with the sessions.

**FIGURE 3:**
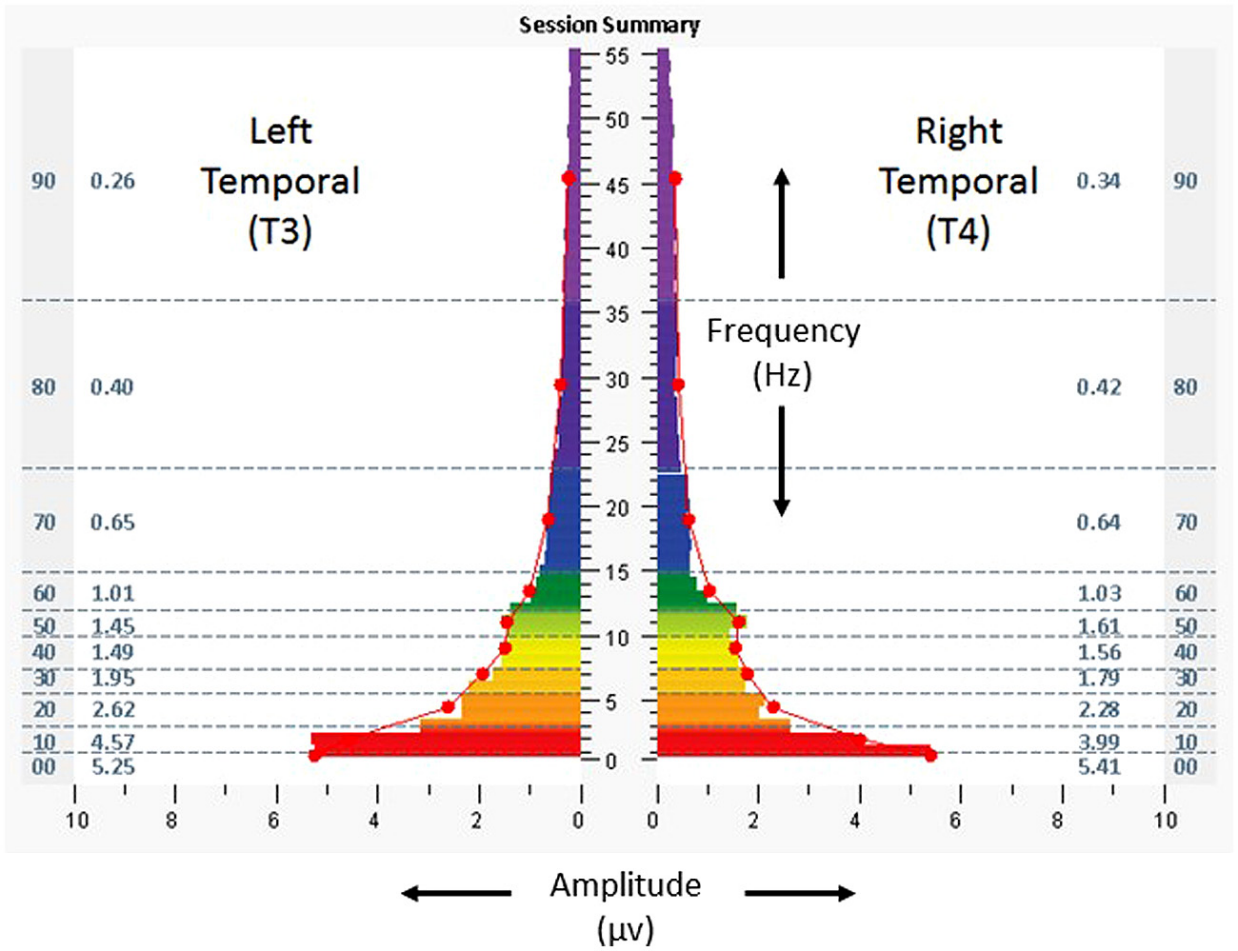
**Spectrographs of left and right temporal lobe brain electrical activity for 29-year-old US military special operations officer (Case 1), during penultimate session of neurotechnology intervention, penultimate minute**. Data reflect subject’s brain activity with eyes closed, at rest, while listening to audible tones. See **Figure 2** legend for detailed explanation of data elements.

**Figure 3** shows a one-minute snapshot of temporal lobe (again T3/T4) brain electrical activity from the penultimate minute of a protocol during the penultimate session, to illustrate the movement towards symmetry which had begun early in the course of sessions (less than 5% asymmetry in higher frequencies). At exit, the subject reported having slept 6 h the preceding night, and scores for the ISI, CES-D, and PCL-C decreased to 19, 22, and 60, respectively. His written comments were that “[I have] discontinued all prescription medication, started sleeping with steady improvement, have reduced pain, increased focus and concentration, and [have had] an improved dynamic with anxiety and depression.” After completing the sessions, the resting heart rate, SDNN, and BRS were 61 bpm, 83.6 ms, and 24.1 ms/mmHg, respectively.

### CASE 2: RIGHTWARD TEMPORAL LOBE BRAIN ELECTRICAL ASYMMETRY IN A FEMALE UNIVERSITY STUDENT WITH PERSISTING POST-CONCUSSION SYMPTOMS INCLUDING DEPRESSION

A 23-year-old woman, a graduate student at a local university, enrolled in the same research study referenced in Case 1, due to persisting post-concussion symptoms. She played soccer and suffered from five concussions during a six month period at age of 13. She then suffered additional, non-athletic concussions at 10 and 5 months prior to enrollment in the study, due to a fall and a mishap while dancing, respectively. She reported persisting headaches and dizziness as primary complaints, was unable to exercise at all, and was in the process of dropping out of graduate school since she was not able to read, study, and learn as needed. She mentioned having migraines during high school but denied other medical problems. She had started amitriptyline (25 mg nightly) three days prior to enrollment in the study but discontinued that medication upon beginning the intervention. Her other medications were oral contraceptives, sumatriptan (25 mg tablet as needed migraine), and ibuprofen (prn).

**FIGURE 4:**
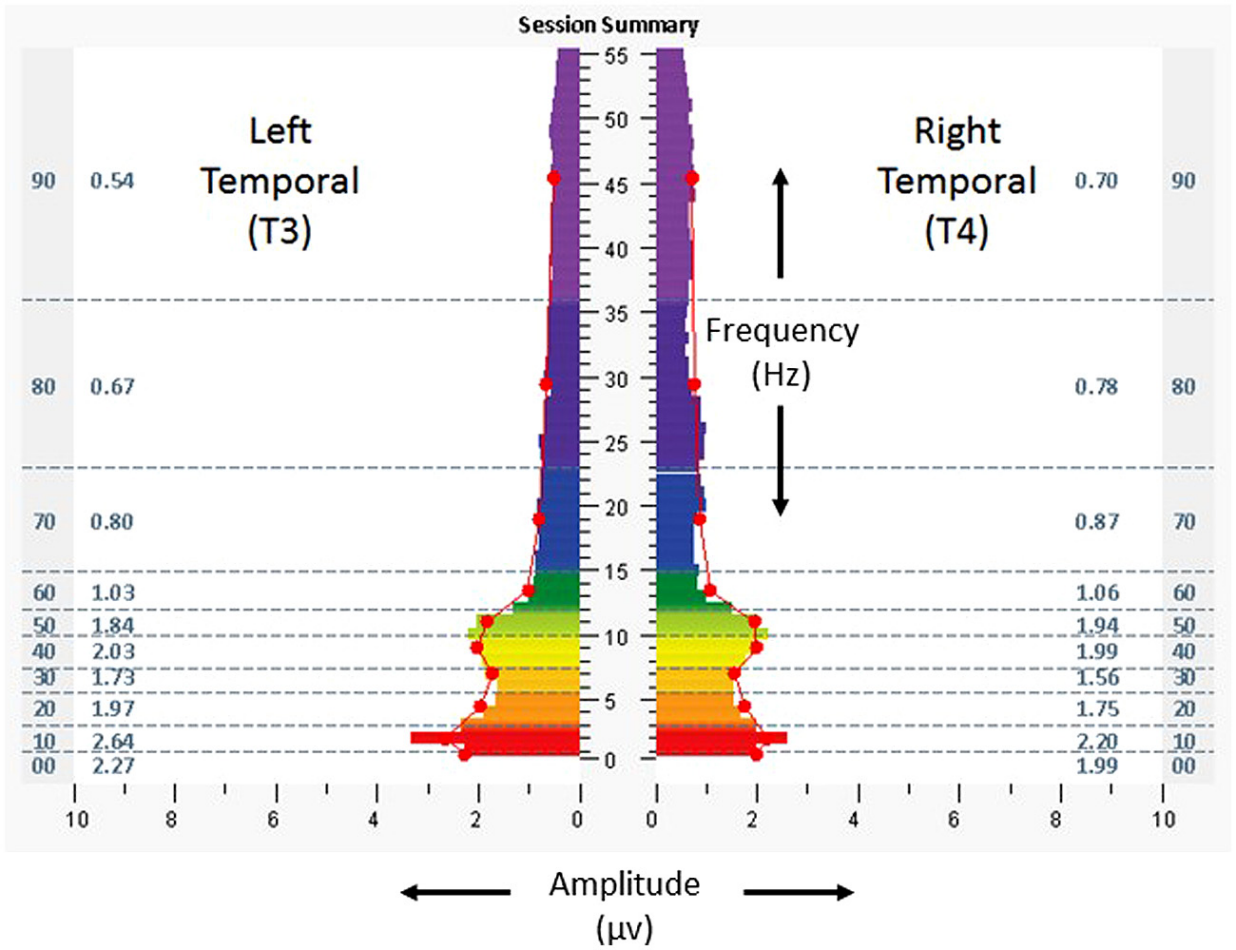
**Spectrographs of left and right temporal lobe brain electrical activity for 23-year-old woman with history of persisting post-concussion symptoms (Case 2), before undergoing neurotechnology intervention for auto-calibration of neural oscillations**. Data were collected from T3 and T4 in 10–20 system, with frequency (Hertz, Hz, vertical axis), plotted against amplitude (microvolts, µv, horizontal axis). See **Figure 2** legend for detailed explanation of data elements.

The baseline assessment at T3/T4, eyes closed (**Figure 4**), revealed T4 dominance (15–29%) in the highest frequencies. Scores for the ISI, CES-D, and PCL-C were 5, 31, and 22. Resting heart rate, SDNN, and BRS were 79 bpm, 55.5 ms, and 13.5 ms/mmHg. She received 23 HIRREM sessions over 34 days and reported no adverse events.

**FIGURE 5:**
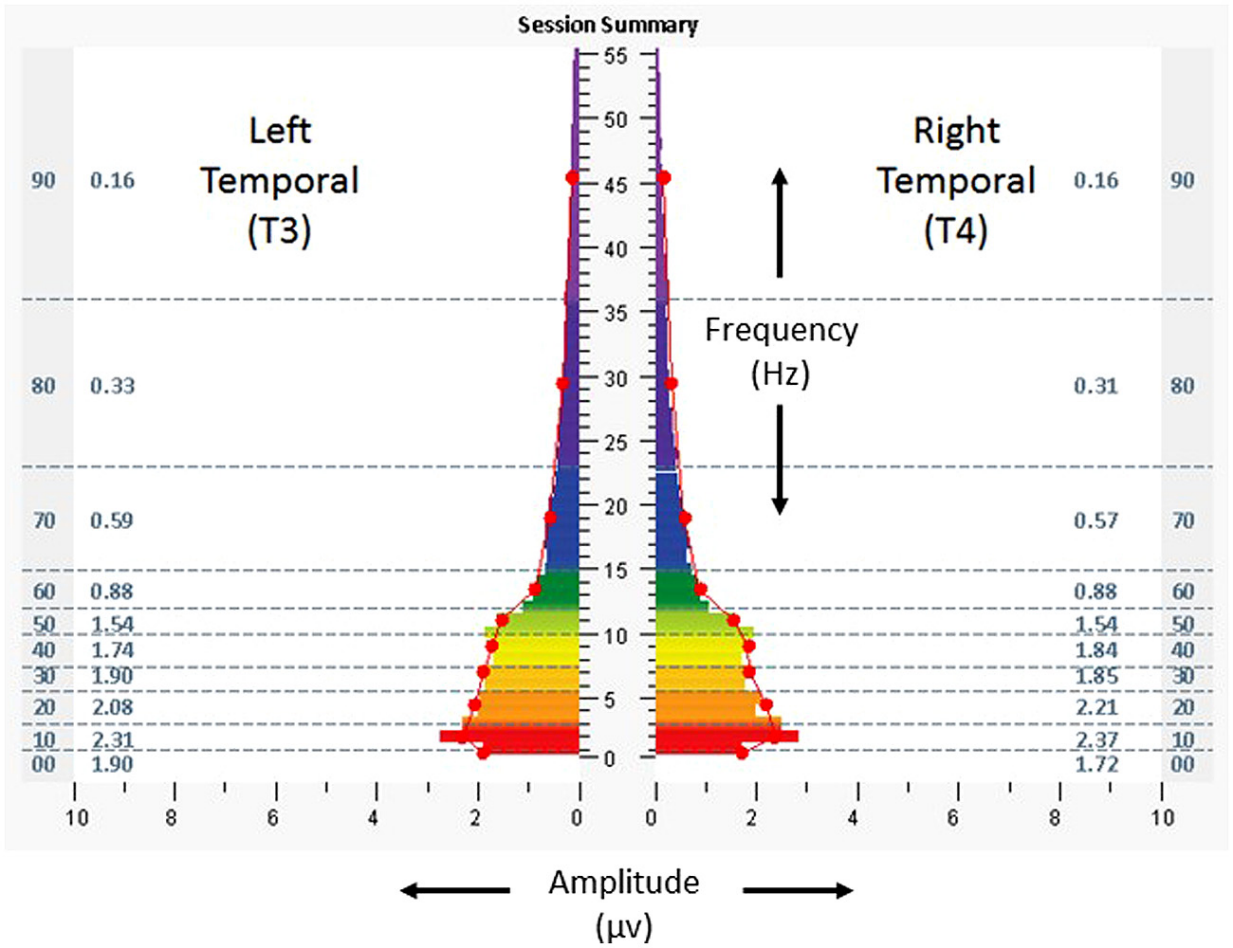
**Spectrographs of left and right temporal lobe brain electrical activity for 23-year-old woman (Case 2), during penultimate session of neurotechnology intervention, penultimate minute**. Data reflect subject’s brain activity with eyes closed, at rest, while listening to audible tones. See **Figure 2** legend for detailed explanation of data elements.

**Figure 5** shows temporal lobe brain electrical activity (eyes closed) from the penultimate minute of a protocol from the penultimate intervention session, with asymmetry reduced to 3–6% in the direction of T3, in the same frequency ranges. During and following the sessions, she reported that she was able to engage in more activities including walking, reading, and watching movies. She also reported improved mood, fewer headaches, increased stamina, better appetite, and improved quality of sleep. Scores for the ISI, CES-D, and PCL-C were 3, 9, and 19. Resting heart rate, SDNN, and BRS were measured at 68 bpm, 83 ms, and 35.7 ms/mmHg.

### COMMENT ON CASE ILLUSTRATIONS

The above cases illustrate asymmetries of brain electrical activity measured at temporal scalp locations. In accordance with our findings that brain electrical asymmetry measured at this location appears to allow assessment of relative sympathetic or parasympathetic activation (Tegeler et al., 2013), subjects reported symptoms related to a number of clinical issues that can manifest as dysregulation of arousal, including insomnia, depressiveness, post-traumatic stress, and pain. McGrath et al. (2013) found that increased right anterior insula activation helped predict treatment response in major depression, and it has been suggested that the asymmetry reported in that study may have been due to differences in autonomic arousal that are detectable through surface measures of brain electrical activity (Tegeler et al., 2014). Traumatic brain injury itself is well appreciated to be associated with autonomic dysregulation (Hilz et al., 2011). For both subjects, use of a noninvasive neurotechnology for auto-calibration of neural oscillations was associated with shifting of temporal lobe brain electrical activity toward greater symmetry, especially in higher frequency ranges. Both subjects reported significant reductions in clinical symptom inventories and overall improvement in functionality.

Cardiovascular measures were consistent with predictions of the BHAM and its incorporation of polyvagal theory. In Case 1, the subject with leftward (T3) dominance was found to have increased resting heart rate after completing the intervention, possibly reflecting release from the “hold” of a parasympathetic freeze state, allowing increased sympathetic activation. In Case 2, the subject with rightward (T4) dominance was found to have decreased resting heart rate post-intervention, possibly reflecting increased functional parasympathetic inhibition from the left hemisphere. In both subjects, heart rate variability (reflected as higher SDNN values) was increased after the intervention, possibly reflecting greater capacity of the myelinated vagus to maintain calm and emotionally well-regulated states as would be predicted by polyvagal theory. Whereas both heart rate and heart rate variability are known to be under vagal regulation (Task Force for the European Society of Cardiology, and the North American Society of Pacing Electrophysiology, 1996), the dissociation of the direction of changes in these two variables for the subject in Case 1 is consistent with recovery of a higher level of autonomic functioning as conceived by polyvagal theory. That is to say, the subject may have demonstrated a shift away from unmyelinated toward myelinated vagal activity, permitting both increased resting heart rate and increased heart rate variability.

We do not presume that the epochs of temporal lobe brain electrical activity shown in the figures are exhaustive and unqualified indicators of ANS management. In the first place, other cortical regions may be of interest. Foster et al. (2008) reported a correlation between frontal asymmetry and heart rate, while other investigators have reported null findings when testing for correlation between heart rate variability and frontal asymmetry (Chang et al., 2012; Balle et al., 2013) or parietal asymmetry (Balle et al., 2013). The possibility cannot be excluded that medication or supplement effects (including changes to regimens) contributed to the brain electrical activity findings in these case studies. Differences in asymmetry between the states of eyes closed and eyes open (at task) may be of particular interest, given that both spectral and topographical aspects of brain electrical activity are known to change between those states (Barry et al., 2007). Moreover, the comparison of brain electrical activity “snapshots” from the assessment and the penultimate intervention session is not meant to imply a linear transition from dominant asymmetry to symmetry. Conceptually, the neurotechnology provided to these subjects is allostatic (“stability through change”) in its intention (Sterling, 2012), aimed to facilitate brain activity to become “un-stuck” from maladaptive set-points (Gerdes et al., 2013). Though the end result tends to be greater hemispheric symmetry and more optimality of ratios between low and high frequencies, every recipient is recognized to demonstrate unique and complex neural oscillatory patterns throughout their process. Whether the BHAM may be better defined by the addition of asymmetry measures for regions other than the temporal lobes, to what degree asymmetry in key regions may be influenced by the state of eyes closed or eyes open or concurrent medication use, and the temporal and spectral dynamics of measured asymmetry (including their reproducibility) over the course of an intervention, are all questions for further study.

Being uncontrolled case examples and in consideration of the variables involved, the above case examples are adduced not as proof of the BHAM but rather as illustrations of its application and as preliminary explorations of phenomena that may be meaningful targets for ongoing study. The neurotechnology used in these case studies has also been associated with reduction of insomnia and depressive symptoms in a pilot clinical trial (Tegeler et al., 2012), but to our knowledge there has otherwise been little attempt to leverage hemispheric lateralization of autonomic management for clinical purposes. In trained cyclists, Okano et al. (2013) have reported that transcranial direct current stimulation over left temporal cortex may modulate sensory perception of effort through delay of parasympathetic withdrawal, to permit increased exercise performance.

We propose that the clinical improvements reported by these subjects may be understood in a way that is highly convergent with the imperative for new frameworks to advance mental health sciences. Specifically, we suggest that these subjects’ symptom clusters, diagnostically differentiated under the schema of the Diagnostic and Statistical Manual but physiologically related under the BHAM, exemplify the need to reconceptualize mental health as the integrated expression of core and interlocked modules of brain-behavior functionality. The NIMH has been vocal in directing mental health researchers to view individuals in such brain-functional terms, rather than through checklists of behavioral and symptom clusters that are compared to population averages (Cuthbert and Insel, 2013). The NIMH RDoC (Research Domain Criteria) initiative has preliminarily identified arousal, positive valence, negative valence, cognitive systems, and social processes as being five core brain-behavioral domains which are operative in both health and disease, and which may serve as more biologically valid units of analysis for future progress in mental health research. We propose that the BHAM is a promising vehicle for fresh efforts in the direction of RDoC and related endeavors.

## DISCUSSION

The present paper has proposed a BHAM that may explain and predict a range of phenomena related to traumatic stress and arousal, mediated partially through relatively autonomous drives toward autonomic auto-calibration. The model proposes that relative symmetry in activity of hemispheric brain regions responsible for autonomic management represents a state of relative optimality in autonomic functioning, whereby sympathetic and parasympathetic functionalities fluctuate naturally and are adaptive for the ongoing needs and changing circumstances of life. Rightward dominant asymmetry in activity of brain regions responsible for autonomic management may reflect a state of sympathetic mobilization which may develop as an adaptive response to a given context, but is likely to be maladaptive if it is persistent despite changing and especially non-threatening environments. Leftward dominant asymmetry in the same brain regions may be indicative of a parasympathetic freeze mode, also adaptive for certain contexts, but also likely maladaptive if persistent. Exposure of an individual to varying degrees and types of traumatic stress, and recovery from the associated traumatic states, produces (or reflects) processes of autonomic auto-calibration toward and away from varying degrees of dominant rightward or leftward asymmetry.

The BHAM concept of autonomic auto-calibration proposes to explain the increased risk for behavioral disturbances (including conduct disorder and substance abuse) among individuals with a history of traumatic stress. For an individual with a history of severe stress or trauma leaving them in a parasympathetic freeze state, compensatory high-arousal behaviors may represent options for *feeling*, despite being at odds with accepted societal norms. Those in a state of traumatic high arousal may be at risk for compensatory behaviors for arousal attenuation, including substance abuse and medication dependence. The model does not imply that relatively autonomous drives for autonomic auto-calibration are not subject to regulation by the prefrontal cortex.

We have illustrated potential interventional implications of the BHAM through case examples of a military veteran with traumatic stress, and a university student with persisting post-concussive symptoms, both of whom had asymmetries of temporal lobe brain electrical activity, and both of whom experienced symptom reduction after using a noninvasive technology designed for auto-calibration of neural oscillations. The subjects’ improvements in arousal-related symptom clusters that cut across diagnostic categories appear to exemplify the value of the RDoC framework proposed by NIMH. Furthermore the traumatic stress history incorporated by the BHAM reflects a sensitivity to neurodevelopmental trajectories that is an advantage of RDoC (Cuthbert, 2014).

Many questions can be asked of the model, to confirm its validity or to explore potential mechanisms or ramifications. We consider that data-collection paradigms based on scalp measures of brain electrical asymmetry are likely to be productive, especially because of their high temporal resolution and ease of implementation, permitting serial measures. Both those advantages may be critical with respect to the capacity of the brain – and the ANS – to shift activity patterns quickly, in the context of rapidly changing and newly anticipated needs. In contrast, some of the core advantages of more complex experimental methodologies, especially their high spatial resolution, are of less consequence if a key parameter of interest is instantaneous hemispheric activation asymmetry.

Within the paradigm of brain electrical measures, it may be asked if there are particular scalp locations most likely to produce reliable indications of autonomic signaling. With respect to the measured signals themselves, studies could be carefully designed to tease apart whether they represent efferent or afferent signals or both, or even one rather than the other depending on the instantaneous context. Spectral components of brain electrical signals may also hold meaningful information. Just three of the testable hypotheses that derive from the BHAM include the following. Nominally healthy and trauma-free individuals who experience an acute (but not overwhelming) stressor, will have greater rightward hemispheric asymmetry in brain regions responsible for autonomic management and greater peripherally measured heart rate, than matched controls who do not experience the stressor. Individuals who have a history of severe or extended exposure to stress or trauma, or children with a history of severe childhood trauma and evaluated to be callous-unemotional, will be more likely to demonstrate left-hemispheric autonomic asymmetry, and they will have on average a lower resting heart rate, than matched controls. In cases of either rightward or leftward asymmetry, it may be hypothesized that degree of asymmetry will correlate with the magnitude of heart rate differences. Third, one may hypothesize that use of interventions to support greater hemispheric symmetry in brain regions responsible for autonomic management will be associated with more optimal autonomic regulation and associated subjective and behavioral improvements.

If the BHAM is valid, then questions should be raised about its generalizability. A new and non-trivial insight about ANS functioning should have new and non-trivial consequences, given the pervasive and critical role of the ANS across organ systems and behaviors (Rees, 2014). It may be that polyvagal theory represented the beginning of a *paradigm shift* for understanding the ANS. And although the phrase “paradigm shift” is now often used loosely to refer to any subjective shift of perspective, we use the phrase in a manner consistent with its original use by the historian and philosopher of science Kuhn (1962). A scientific paradigm shift begins when normal science is met with an anomaly. In the case of the ANS, understanding of the stress-buffering role of the parasympathetic nervous system did not cohere with understanding about the potential lethality of neurogenic bradycardia. Polyvagal theory explained that anomaly by articulating two different forms of vagal activity, especially identifying distinct features of the parasympathetic freeze state, and proposing that autonomic activity is expressed in a hierarchical way in accordance with environmental context and individual variations. New scientific paradigms require new tools and procedures for collecting and interpreting empirical data, and we suggest that measurement of brain electrical asymmetry may represent a productive new approach for autonomic neuroscience. If a paradigm shift for assessment and intervention on autonomic dimensions of brain-behavior relationships is now in the making, we hope for the BHAM to support productive explorations of the new worldview.

## Conflict of Interest Statement

Dr. Sung W. Lee and Mr. Lee Gerdes are employees and shareholders of Brain State Technologies LLC, developer of the neurotechnology described in the case illustrations. Ms. Catherine L. Tegeler, Dr. Hossam A. Shaltout, and Dr. Charles H. Tegeler declare that they have no commercial or financial relationships that could be construed as a potential conflict of interest with respect to this article.
